# Hypergraphs and Cellular Networks

**DOI:** 10.1371/journal.pcbi.1000385

**Published:** 2009-05-29

**Authors:** Steffen Klamt, Utz-Uwe Haus, Fabian Theis

**Affiliations:** 1 Max Planck Institute for Dynamics of Complex Technical Systems, Magdeburg, Germany; 2 Institute for Mathematical Optimization, Faculty of Mathematics, Otto-von-Guericke University Magdeburg, Magdeburg, Germany; 3 Institute for Bioinformatics and Systems Biology, Helmholtz Zentrum München—German Research Center for Environmental Health, Neuherberg, Germany; 4 Max Planck Institute for Dynamics and Self-Organization, Göttingen, Germany; ETH Zürich, Switzerland

## Background

The understanding of biological networks is a fundamental issue in computational biology. When analyzing topological properties of networks, one often tends to substitute the term “network” for “graph”, or uses both terms interchangeably. From a mathematical perspective, this is often not fully correct, because many functional relationships in biological networks are more complicated than what can be represented in graphs.

In general, graphs are combinatorial models for representing relationships (edges) between certain objects (nodes). In biology, the nodes typically describe proteins, metabolites, genes, or other biological entities, whereas the edges represent functional relationships or interactions between the nodes such as “binds to”, “catalyzes”, or “is converted to”. A key property of graphs is that every edge connects two nodes. Many biological processes, however, are characterized by more than two participating partners and are thus not bilateral. A metabolic reaction such as A+B→C+D (involving four species), or a protein complex consisting of more than two proteins, are typical examples. Hence, such multilateral relationships are not compatible with graph edges. As illustrated below, transformation to a graph representation is usually possible but may imply a loss of information that can lead to wrong interpretations afterward.

Hypergraphs offer a framework that helps to overcome such conceptual limitations. As the name indicates, hypergraphs generalize graphs by allowing edges to connect more than two nodes, which may facilitate a more precise representation of biological knowledge. Surprisingly, although hypergraphs occur ubiquitously when dealing with cellular networks, their notion is known to a much lesser extent than that of graphs, and sometimes they are used without explicit mention.

This contribution does by no means question the importance and wide applicability of graph theory for modeling biological processes. A multitude of studies proves that meaningful biological properties can be extracted from graph models (for a review see [1]). Instead, this contribution aims to increase the communities' awareness of hypergraphs as a modeling framework for network analysis in cell biology. We will give an introduction to the notion of hypergraphs, thereby highlighting their differences from graphs and discussing examples of using hypergraph theory in biological network analysis. For this Perspective, we propose using hypergraph statistics of biological networks, where graph analysis is predominantly used but where a hypergraph interpretation may produce novel results, e.g., in the context of a protein complex hypergraph.

Like graphs, hypergraphs may be classified by distinguishing between undirected and directed hypergraphs, and, accordingly, we divide the introduction to hypergraphs given below into two major parts.

## Undirected Hypergraphs

An *undirected hypergraph* ***H*** = (*V*,*E*) consists of a set *V* of vertices or nodes and a set *E* of hyperedges. Each hyperedge *e*∈*E* may contain arbitrarily many vertices, the order being irrelevant, and is thus defined as a subset of *V*. For this reason, undirected hypergraphs can also be interpreted as *set systems* with a ground set *V* and a family *E* of subsets of *V*. If no hyperedge is a subset of another hyperedge, ***H*** is also called a *Sperner* hypergraph, or *clutter*.

**Figure 1:**
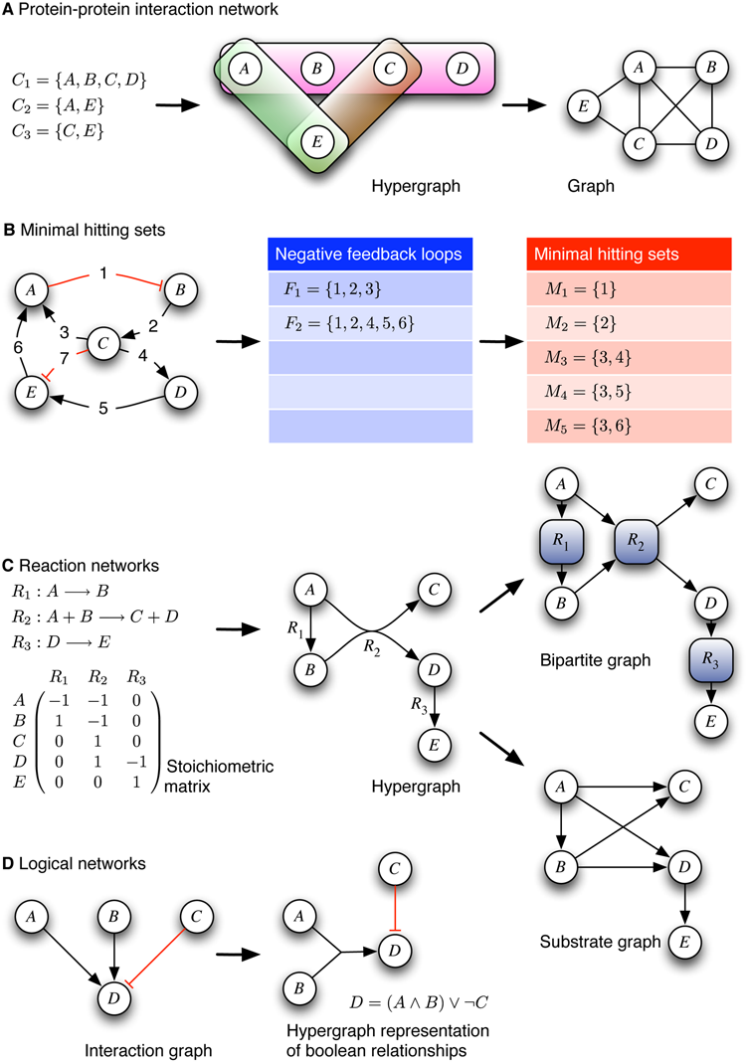
Examples of undirected (A,B) and directed (C,D) hypergraphs arising in the context of biological networks analysis. Detailed explanations are given in the text.

Undirected *graphs* are special cases of hypergraphs in which every hyperedge contains two nodes (i.e., has a cardinality of two). Protein–protein interaction (PPI) networks provide a nice example illustrating the differences that may arise in modeling biological facts with graphs and hypergraphs. Various technologies for measuring protein interactions have been developed, but we concentrate here on data obtained, e.g., by tandem affinity purification (TAP, [2],[3]) delivering protein complexes (with possibly more than two partners) instead of direct binary interactions. A small-scale example mimicking experimental data derived by TAP is shown in Figure 1A (left). TAP data naturally span a hypergraph: We have a ground set of proteins and a set of complexes, which themselves represent subsets (hyperedges) of the ground set of proteins. One method for drawing undirected hypergraphs is shown in Figure 1A (middle). Hypergraphs are often projected onto graphs, losing some information but making their drawing easier and their analysis amenable to the huge corpus of methods and algorithms from graph theory. A typical graph representation of our example is shown in Figure 1A (right) (another way to convert hypergraphs to graphs will be shown below). This representation still captures the information on pairs of proteins that occurred together in a complex; however, in contrast to the hypergraph, the complexes themselves cannot be reconstructed from this figure. This may lead to different results when computing network properties such as the *k*-core, a measure that is often used to identify the core proteome [4],[5]. In a graph, the *k*-core is the maximal node-induced sub-graph in which all nodes have a degree (defined as the number of edges a node participates in) equal to or larger than *k*. The maximum core of a graph corresponds to the highest *k* where the graph has a non-empty *k*-core. The maximum *k*-core of the graph in Figure 1A is a 3-core consisting of the nodes {A,B,C,D}. A similar definition of a *k*-core can be defined for (Sperner) hypergraphs, where *k* corresponds to the number of hyperedges each node participates in [5]. The maximum *k*-core of the hypergraph in Figure 1A is a 2-core consisting of {A,C,E}. Thus, as one would intuitively expect, the maximum *k*-core of the hypergraph ranks A, C, and E as most important—in contrast to the graph model, whose maximum *k*-core would weight B and D stronger than E.

Another application of undirected hypergraphs is *minimal hitting sets* (MHSs), also known as generalized vertex covers or hypergraph transversals [6],[7]. For example, in a given hypergraph model of a PPI network, an interesting problem related to experimental design [5] is to determine minimal (irreducible) subsets of bait proteins that would cover or “hit” all complexes in a minimal way; i.e., no proper subset of an MHS would hit all complexes. In Figure 1A, the corresponding MHSs would be {A,E},{A,C},{B,E},{C,E},{D,E}. MHSs are also relevant for computing intervention strategies [8],[9]. An example: Assume that a network (which is in our example a directed graph) contains feedback loops, and a given selection of them is to be disrupted with appropriate interventions. This is equivalent to computing MHSs in a hypergraph, where *V* is the ground set of interactions (here: edges) and *E* is the set of targeted feedback loops, i.e., each hyperedge contains the involved interactions of one feedback loop. Figure 1B shows a simple interaction graph (left) containing three feedback loops, thereof two being negative (Figure 1B, middle). There are five MHSs for disrupting the negative feedback loops: Two of them remove only one edge, whereas the other three cut two edges. Even though they require two interventions, the MHSs {3,4} and {3,5} will be preferred if the only positive feedback loop in the network, constituted by edges 1,2,7,6, is to be kept functional. In a very similar way, one may compute MHSs of a “target set” of *elementary modes*, revealing intervention strategies in metabolic networks [8],[10].

Hypergraphs are also closely related to the concept of *independence systems*. An independence system ***I*** = (*V*,*U*) is a collection *U* of subsets of a ground set *V* in which for each set *u*∈*U* all subsets of *u* are part of the collection. Any Sperner hypergraph ***H*** = (*V*,*E*) can be extended to an independence system ***I*** = (*V*,*U*) in which *V* is still the set of vertices and *U* contains all hyperedges of *E* plus all subsets of these hyperedges. The hyperedges of the original hypergraph are then the maximal independent sets (also called bases) of the independence system ***I***. For example, the family of sets of the independence system induced by the protein complex hypergraph in Figure 1A would contain the three protein complexes (the maximal independent sets) plus all subsets of each complex. Consider now the following problem: Each protein is assigned a weight representing, for instance, the molecular weight of the protein. We could ask for the complex of maximal weight. Can we find such a complex without examining all complexes, i.e., all maximal independent sets? If not, how good are approximations that we can find quickly? These questions can be answered by the theory of independence systems using methods from discrete optimization and combinatorics [11],[12]. The most prominent type of independence system is that of a *matroid* [13]. Optimization problems on matroids are of low complexity because the simple *greedy algorithm* (taking in each step the locally optimal choice) always finds a globally maximal independent set. Coming back to the optimization problem of finding the heaviest protein complex in Figure 1A, assume the (molecular) weights are as follows: A = 1, B = 2, C = 3, D = 4, E = 5. A greedy strategy (operating on the vertices) would first select protein E because it has the highest weight. This reduces the search space to complex *C_2_* and *C_3_*. For the next protein we choose C because its molecular weight is larger than that of A. The algorithm finishes at that point as it has found a maximal independent set (complex *C_3_*) whose weight is 8, which is apparently not the optimum (note that this is not due to the larger size of complex *C_1_*; choosing A = 8, B = 1, C = 1, D = 9, E = 8, the greedy algorithm would deliver the four-protein complex *C_1_*, although the true optimum is then the two-protein complex *C_2_*). The reason that the greedy algorithm fails in this simple example is that the independence system spanned by the complex hypergraph is not a matroid.

Given how frequently greedy-type algorithms on hypergraphs are applied as heuristics in practice, it appears important to study the deviation of the hypergraph under consideration from being a matroid [13]. A recent study on algorithms for measuring phylogenetic diversity underlines this point [14].

## Directed Hypergraphs

The definition of *directed hypergraphs* is similar to undirected hypergraphs, ***D*** = (*V*,*A*), but each hyperedge *a*∈*A*—here also called hyperarc—is assigned a direction, implying that one has to define where it starts and where it ends. Directed hypergraphs allow us to connect *several* start nodes (the tail 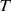) with *several* end nodes (the head *H*). A hyperarc is thus defined as *a* = (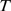,*H*) with 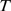 and *H* being subsets of the vertices *V*. Again, directed graphs are special cases of directed hypergraphs where both 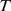 and *H* contain exactly one node limiting their scope to 1∶1 relationships. In contrast, directed hypergraphs can represent arbitrary n:m relationships.

Typical examples are (bio)chemical reactions, which are often bi-molecular, such as the example A+B→C+D. The tail 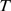 of this hyperarc consists of the reactants A and B, whereas the head *H* contains the product C and D. However, for an exact description of stoichiometric reactions we need to include the stoichiometric coefficients (which can be different from unity) in the hypergraph model. For this purpose, one adds into each hyperarc two functions 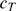: 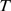
→**N** and *c_H_*: *H*→**N**, assigning the stoichiometric coefficients for the nodes in 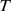 and *H*, respectively. Each hyperarc *a* then reads *a* = (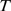, 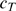, *H*, *c_H_*). This completes the description of a stoichiometric network, which is in practice often conveniently described by a stoichiometric matrix (Figure 1C): The columns correspond to the reactions, i.e., hyperarcs, and the rows to the nodes, i.e., metabolites with their stoichiometric coefficients [15]. Reactants can be distinguished from the products by the negative sign at their stoichiometric coefficients.

Directed hypergraphs can be drawn as shown in the example in Figure 1C. For simplifying drawing and analysis, directed hypergraphs are often converted (Figure 1C, right) either to directed substrate graphs (similar to the graph in Figure 1A) or to directed bipartite graphs. In the latter, both reactions and metabolites are represented as two different types of nodes, and edges exist only from metabolites to reactions, or vice versa. In contrast to the simple graph projection used in the substrate graph, the bipartite graph still reflects the original information from the hypergraph. This representation can be used to determine a number of relevant topological network properties using graph-theoretical techniques [16]. However, even in bipartite graphs, graph-theoretical methods may not be appropriate when analyzing functional properties that require an explicit consideration of the AND connections between reactants and products. For example, as one can easily verify, removing reaction R1 from the reaction network in Figure 1C implies that a continuous production of E from A alone would not be possible anymore. However, a path from A to E still exists in the bipartite graph (via nodes R2, D, and R3), which might suggest that this was still possible. Techniques of the popular constraint-based analysis of metabolic networks [15] directly operate on the stoichiometric matrix and therefore take the hypergraphical nature of metabolic networks explicitly into account. Using a prominent example from the central metabolism (production of sugars from fatty acids), a recent contribution illustrates that non-functional pathways might be detected in metabolic networks when paths in the underlying graph representation are interpreted as valid routes [17]. A widely used concept for pathways in hypergraphical reaction networks is based on *elementary (flux) modes*, which are minimal functional sub-networks able to operate in steady state [18]. Elementary modes are better suited for studying functional aspects of metabolic networks than simple paths in the graph representation. However, it comes at the expense of higher computational efforts. For example, the calculation of the elementary mode with the smallest number of reactions involved is much harder (NP-hard [19]) than the easy problem of computing shortest paths in graphs. Furthermore, some problems can be safely studied in the graph representation. An example from Figure 1C: The shortest “influence” path along which a perturbation in the concentration of metabolite A can spread over the network and affect the concentration of node E involves two steps (reactions R2 and R3) and can be deduced from both graph representations. Even if reaction R1 is absent, this path would be valid if we assume that the concentration of B is non-zero at the beginning. What would not be possible with R2 and R3 alone, as discussed above, is a *continuous* production of E when A is provided as a substrate.

Another application of directed hypergraphs in computational biology is the representation of logical relationships in signaling and regulatory networks. Interaction graphs (signed directed graphs) are commonly used topological models for causal relationships and signal flows in cellular networks. For example, in Figure 1D, species A and B have a positive and C a negative influence on the activation level of D. However, due to the 1∶1 relationships, we cannot decide which combinations of input signals of D will eventually activate D itself. With additional information, a refined hypergraph representation might be constructed as in the right part of Figure 1D: The hyperarc connecting A and B with D expresses a logical AND, whereas the (simple) red hyperarc from C to D indicates an alternative way to activate D, namely if the inhibiting species C is not active. Hence, this hypergraph expresses the Boolean function “D gets activated if A AND B are active OR if C is inactive”. In fact, any Boolean network can be represented by a directed hypergraph [9], which can be advantageous when analyzing biologically relevant network properties [9],[20],[21]. Again, a correct analysis of network function and dysfunction, e.g., which knock-out combinations guarantee an inactivation of D in Figure 1D, requires the explicit consideration of AND relationships properly captured by hypergraphs.

## Algorithmic Considerations

The concept of hypergraphs provides such a rich modeling framework that algorithms necessarily will be problem-specific, and will differ in complexity from similar algorithms for graphs. Clearly, since graphs are special cases of hypergraphs, algorithms for hypergraphs are at least as hard as its specialized implementations in the graph case. Generally, when discussing algorithms in graphs and hypergraphs, one has to distinguish between two types of problems. The first type encompasses algorithms determining a *particular* (e.g., optimal) solution. One example, as noted above, are shortest-path algorithms for graphs that are of low complexity (and thus applicable in large-scale networks) and which can also be used to find the connected components or to determine spanning trees in a hypergraph. This is due to the fact that the graph representation as in Figure 1C captures all necessary information for these questions. If hyperedges are weighted, however, the shortest-path problems are hard ones in general, unless assumptions about the structure of the edge weight function are made: If each edge is weighted by its cardinality, the shortest-path problem is NP-hard, but if the weight function is additive, the problem can be solved using a modified Dijkstra algorithm [22]. On the other hand, problems that are computationally easy for graphs can be hard for hypergraphs: Finding a maximal matching in a bipartite graph, i.e., determining a set of edges with maximal weight so that each node is contained in exactly one of the edges, is polynomial time–solvable. Even checking whether a hypergraph is bipartite, i.e., can be partitioned into two sets of nodes so that no hyperedge is contained in either of them, is NP-hard [23].

The second type of problem is *enumeration problems* such as computing all paths and cycles in a graph or all minimal hitting sets in a hypergraph. These problems typically require enormous computational effort and are often limited to networks of moderate size. For example, the hardness of computing the minimal hitting sets (transversal of a hypergraph) is an open question in complexity theory [11]. The theoretically fastest currently known algorithm is quasi-polynomial [24], used successfully, e.g., in [12], whereas variants of Berge's method [6] are often faster in practice [10]. In general, it turns out that the particular topology of cellular networks renders enumeration problems often feasible where one would expect infeasibility in random networks with comparable size (see, e.g., [10],[25]).

## Network Statistics in Hypergraphs

With the increasing availability of large-scale molecular interaction graphs such as PPI or gene regulatory networks, more and more researchers have begun asking not only for single specific elements of a graph but instead for its statistical properties or significant building blocks. Examples are the neural network of *C. elegans*, which satisfies the small-world property, implying shorter mean shortest paths and higher clustering coefficients than one would expect in random networks [26], and the PPI network of yeast, which may be modeled using a scale-free topology and whose node connectivity is correlated with essentiality of the corresponding protein [27]. Key novelties in these approaches are that properties of the graphs are now interpreted as statistical distributions, which can be correlated with other variables and asked for significance within an appropriate class of random graphs [28],[29]. In the following, we will first shortly outline some existing extensions of graph statistics to hypergraph statistics and corresponding random models and afterward indicate applications in computational biology. We will focus on undirected hypergraphs, although extensions to directed ones are possible.

The degree *d*(*ν*) of a vertex *ν*∈*V* of an undirected hypergraph ***H*** = (*V*,*E*) is the number of hyperedges that contain *ν*. Similarly, the degree *d′*(*e*) of an hyperedge *e*∈***H*** is the number of vertices of that hyperedge. If *G* is a graph, then *d′*(*e*) = *2*. In the more general hypergraph setting, however, we can consider distributions both of vertex and hyperedge degrees. We can ask for mean degrees or more general properties of the distributions. In social network analysis, this has already been done: For instance, an actor–movie hypergraph obeys power-law distributions in both degrees whereas an author–publication hypergraph shows a power law only in the number of co-authored papers, but not in the author degree [30]—which is simply due to the fact that the number of authors on a paper is relatively limited.

The natural next step in defining hypergraph statistics is to correlate vertex and hyperedge connectivity, a major ingredient for determining, e.g., the small-world property known from the graph case [26]. Here, the commonly used graph clustering coefficient may be extended. For this, let 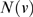 denote the neighborhood of a vertex, which is defined as the set of hyperedges that contain *ν*. Then the (hypergraph) clustering coefficient *cc* defined for a pair of vertices (*u*,*ν*) is given by 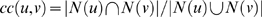, which quantifies overlap between neighborhoods. By analogy, it can be defined for hyperedges as well, and, by averaging over all vertices, a univariate clustering coefficient may be defined. In the author–publication hypergraph, clustering coefficients of both vertices and hyperedges are higher than expected by chance [30]. Another proposal for clustering coefficients in hypergraphs can be found in [31]. In addition to such local measures, we may also ask for global or semi-global properties. A common question in the graph case is to identify clusters, often denoted as communities, within the graph. Various methods have been proposed in this context, with normalized cut [32] and graph modularity [33] being two of the most popular ones, resulting in applications such as the search for modular structures, ideally protein complexes, in PPI networks [34]. The former method has already been extended to hypergraphs [35].

In order to test for significance of certain structures, e.g., network motifs [36] or scaling structures [26],[27], good null models are important. Such null models describe random occurrences of structures. One typically wants to keep some statistics of the network fixed while at the same time randomly sampling from its representational class. This results in the notion of random graphs with certain additional properties such as Erdös-Rényi [37] or Barabási-Albert [38]. Extensions of random models, in particular to hypergraphs, would focus on generative models, which increasingly find applications at least in the graph case [26],[39]. In the context of hypergraphs, first models have already been proposed [40].

**Figure 2:**
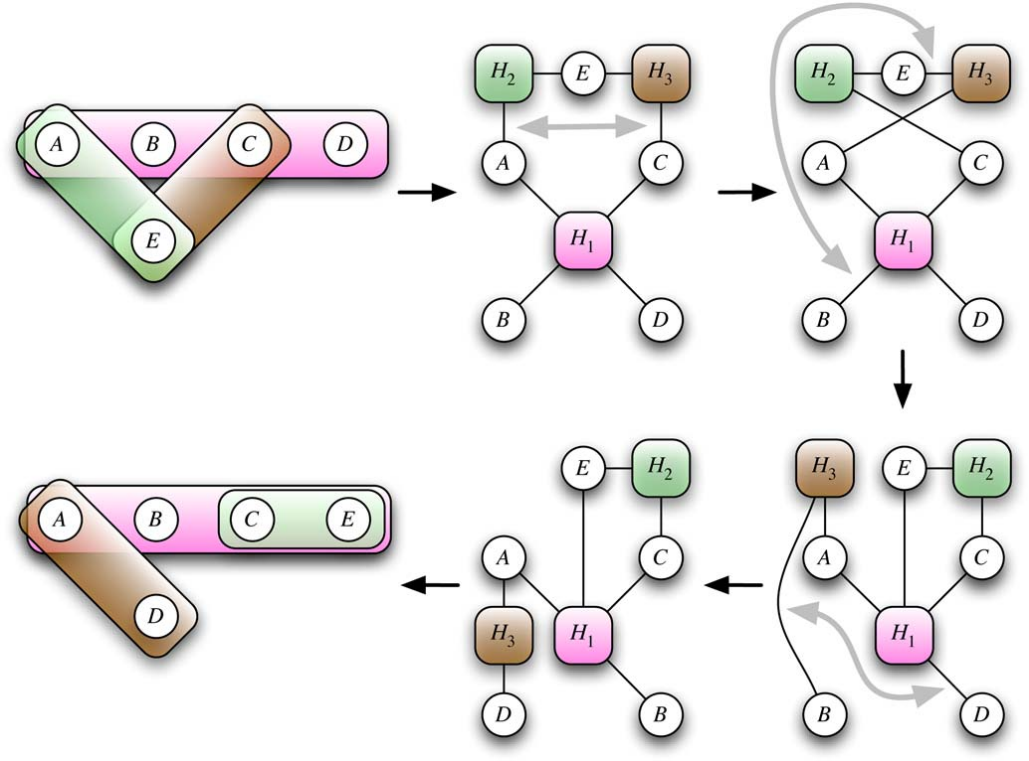
Generating a hypergraph null model by rewiring. Choose two distinct hyperedges and two different vertices contained in either of the two. Then swap them. Clearly this operation keeps both degree distributions fixed. After a certain number of iterations, the thus-generated Markov chain produces independent samples of the underlying random hypergraph with given degree distributions. In the figure, this is illustrated using the in-this-case simpler-to-visualize bipartite version. The gray double-arrows indicate edges to be swapped. Each of the three swaps, (A,H_2_)–(C,H_3_), (B,H_1_)–(E,H_3_), and (B,H_3_)–(D,H_1_), does not change the vertex and edge degrees. Significance analysis of the CORUM protein complex hypergraph was done in [44] using this idea.

What could be potential biological applications of hypergraph statistics? Given the fact that in gene regulatory networks statistical properties are decisive [27], it stands to reason that if one wants to combine two types of regulations or interactions, e.g., gene and microRNA regulation, the resulting hypergraph ought to be analyzed from a hypergraph statistics point of view. Another example is the human–disease network [41], consisting of disease genes and related diseases. Often, analysis and visualization are done on the projected versions, either onto diseases or genes. However, node statistics or motif detection [36] may be performed in the hypergraph itself. The latter is already implemented, e.g., in FANMOD [42], a motif-finding tool ready to deal with n-partite networks. Finally, we want to mention a hypergraph analysis of a mammalian protein complex hypergraph acquired from the CORUM database [43]. The hypergraph shows scale-free behavior in both vertex degree and hyperedge degree distribution [44]. As illustrated schematically in Figure 2, the authors then built a random hypergraph, in which each node and each edge still had the same degrees as in the original hypergraph, but where any higher-order node correlations such as the clustering coefficients were destroyed. By using this hypergraph null model, the authors were able to show that certain large protein complexes with low mean protein length would not be expected by chance. Altogether, hypergraph statistics can be easily applied to, e.g., networks of interactions between nodes of two types, and first examples already show promising results.

## Conclusions

To summarize, hypergraphs generalize graphs by allowing for multilateral relationships between the nodes, which often results in a more precise description of biological processes. Hypergraphs thus provide an important approach for representing biological networks, whose potential has not been fully exploited yet. We therefore expect that applications of hypergraph theory [6],[22] in computational biology will increase in the near future.
